# International Classification of Functioning, Disability and Health in Vocational Rehabilitation: A Scoping Review of the State of the Field

**DOI:** 10.1007/s10926-018-9788-4

**Published:** 2018-06-05

**Authors:** A. H. Momsen, C. M. Stapelfeldt, R. Rosbjerg, R. Escorpizo, M. Labriola, M. Bjerrum

**Affiliations:** 1 Section of Clinical Social Medicine and Rehabilitation, Department of Public Health, Aarhus University, 8000 Aarhus, Denmark; 2 DEFACTUM - Social & Health Services and Labour Market, Central Denmark Region, 8000 Aarhus, Denmark; 3 Department of Rehabilitation and Movement Science, University of Vermont, Burlington, VT USA; 4 Swiss Paraplegic Research, Nottwil, Switzerland; 5 Danish Centre of Systematic Reviews: A Joanna Briggs Institute Centre of Excellence, Department of Medicine and Technology, University of Aalborg, 9000 Aalborg, Denmark; 6 Section of Nursing Science, Department of Public Health, Aarhus University, 8000 Aarhus, Denmark; 7 DEFACTUM, Aarhus University Hospital, MarselisborgCentret, P.P. Oerums Gade 11, 8000 Aarhus, Denmark

**Keywords:** Occupational health services, Rehabilitation, Return to work, Outcome assessment

## Abstract

*Purpose* The purposes of this study were to provide an outline of the existing literature on operationalization of the International Classification of Functioning, Disability and Health (ICF) within vocational rehabilitation (VR) and to explore the ICF utility within VR. *Methods* The process was undertaken in five stages according to a framework of scoping review. Screening and extraction of data were done by two independent reviewers, and data was summarized according to content analysis. *Results* Fifty papers (25 qualitative and 25 quantitative) were included. The operationalization of the ICF was described in four different ways: In total 18 (36%) papers described use of the ICF for structuring information, 8 (15%) for linking information to ICF, 12 (24%) for analysis of results, and 12 (24%) for development of a model. In total 15 (29%) papers described VR interventions involving stakeholders, whereas 32 (62%) were reviews. Forty of the papers described all the ICF components. *Conclusions* The review revealed use of the ICF within the field VR in 50 papers, and in various settings. The ICF framework was most often operationalized for structuring or linking information of functioning. A majority of papers were reviews and involved researchers only, whereas different stakeholders and VR professionals were involved in the interventions. In 40 papers all the ICF components were described, and the ICF was considered a useful tool to inform the VR professionals´ assessment of functioning. However, more research within VR is needed to standardize and ease the use of the ICF.

**Electronic supplementary material:**

The online version of this article (10.1007/s10926-018-9788-4) contains supplementary material, which is available to authorized users.

## Background

**Fig. 1:**
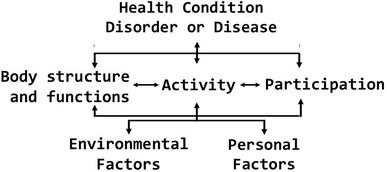
The internation classification of functioning, disability and health (ICF) framework

Work disability is often associated with personal suffering and loss of income, diminished productivity and increased medical and societal costs and can be addressed through vocational rehabilitation (VR) [1]. The essence of VR is promotion of workers’ health in order to enter or return to work (RTW), prevent work disability, and sustain work ability [1–4]. VR professionals have been challenged by different perceptions of health, and researchers argue for a definition of health as a dynamic process of adaptation and self-management [5]. The Organisation for Economic Co-operation and Development (OECD) states that several countries have made efforts to move away from assessing a person’s illness, but instead examining the person’s remaining work capacity [6]. The International Classification of Functioning, Disability and Health (ICF) (See Fig. 1) was approved by the World Health Assembly in 2001 [7], and the ICF framework covers a spectrum of body, personal, and societal aspects of human functioning. Thereby, the ICF captures a comprehensive view of disability relevant to VR, and the integration of “functioning” in VR rather than the traditional biomedical approach, which is in line with the efforts stated by OECD [2]. In VR a comprehensive understanding of the aspects influencing patients’ functioning is important. Thus, the usefulness of the ICF may be demonstrated in VR [8]. The ICF framework has been proposed to offer opportunities to optimize VR for patients by providing a universal conceptual reference to improve communication between different users, such as health care professionals, researchers, and policy-makers.

There are several definitions of VR, e.g. medical, psychological, social and occupational activities aiming to reestablish sick or injured peoples work capacity and prerequisites for returning or entering the labour market, i.e. to a job or availability for a job, 2009 [9]. In 2011, a broader ICF-based definition of VR was introduced: “A multi-professional evidence-based approach that is provided in different settings, services, and activities to working age individuals with health-related impairments, limitations, or restrictions with work functioning, and whose primary aim is to optimize work participation” [2].

A review showed diversity in the ICF contents of the measures used in the literature, and proposed that the ICF and VR interface should be further examined [10]. Knowing more about how and to what extent the ICF framework is applied and has been utilized is suggested important in order to optimize VR interventions for patients [11] and the inter-professional approach in VR processes [8].

The purposes of this review were to provide an outline of the existing literature and to explore the ICF utility within VR. The primary aim was to examine and map the operationalization of the ICF within VR. The second aim was to examine the different VR professionals´ use of the ICF. Ideally a multi-professional, multimodal approach should be used in VR [12].

Within the WHO a number of ICF core sets have been developed in order to make the ICF more applicable for clinical practice. A third aim was to examine to what extent the components of the ICF framework, the VR core set, and other ICF core sets are used within VR. Core sets are lists of essential ICF-categories in specific health conditions and contexts to describe functioning, e.g. a comprehensive and a brief VR core set were developed and validated for interdisciplinary assessment, documentation, and communication in VR [13, 14].

A preliminary search in PROSPERO and PubMed showed no review on the topic, and to our knowledge there are no existing systematic reviews or scoping review on how the ICF is applied within VR.

## Methods

The scoping review was conducted according the methodology conduced in five steps: (1) identifying the research question, (2) identifying relevant studies, (3) study selection, (4) charting the data, and (5) collating, summarizing, and reporting results [15–17].

### Identifying the Research Question

1. How is the ICF operationalized in empirical papers within VR?
2. Who are involved and how does the ICF inform the professionals´ assessment of functioning in VR?
3. Which of the ICF components and core sets are considered when functioning is evaluated in VR?

### Identifying Relevant Studies

A three-step search strategy was conducted [16]. Firstly, initial keywords were identified and secondly all identified keywords and index terms were used to build a comprehensive and specific search strategy for each included database: PubMed, Embase, Scopus, CINAHL, PsycINFO, Swemed+, and PEDro. Thirdly, the search strategies were refined: VR and RTW (MeSH term) in PubMed and other terms, e.g. sick leave, work disability were used as keywords [10], and ICIDH was used as ICF was not a MeSH term until 2012 [18, 19]. The search was performed in collaboration with a research librarian at Aarhus University Library. The search was restricted to papers in English, German, Danish, Swedish, and Norwegian (Online Appendix A).

### Study Selection

Inclusion criteria: ICF or International Classification of Functioning, Disability and Health mentioned in the title or abstract, ICF used in the field of VR research, peer reviewed original papers and reviews, date of publication from January 2001 to May 2016, abstract available, and study populations of working age adults. There were no limitations regarding including reviews and thereby potential overlap of individual papers included in the reviews. There were no context limitations regarding geography or culture, and papers were eligible from any healthcare setting or research setting (e.g. rehabilitation clinic, in-patient or out-patient clinic, hospital, physicians, primary health care, occupational health services, insurance office, and research departments).

Exclusion criteria: papers only mentioning ICF in the abstract, background or discussion, or only mentioning ICIDH or ICIDH-2, overviews, editorials, comments, theoretical papers, text and opinion papers, theses/dissertations, books, and papers on ICF-Children and Youth.

The process of study selection was reported using the PRISMA [20], and eligible studies were screened independently by two reviewers (AM and MB) followed by consensus discussions. The selection was performed in two groups for qualitative papers (AM and RR) and quantitative papers (AM and CMS), respectively.

### Extraction of Data

Study characteristics were extracted from the included studies using a pilot-tested non-software template. The papers were divided in qualitative and quantitative papers according to qualifications of the review team. Two authors extracted study characteristics independently for qualitative papers (AM and RR) and quantitative papers (AM and CMS), respectively. In case of disagreement, the final decision about characteristics was resolved through discussion. The papers were divided in qualitative and quantitative papers according to the data collection method described.

Study characteristics according to The Joanna Briggs Institute Reviewers’ Manual included: first author, publication year, country, setting, study type (intervention yes/no), population, aims, methods, and outcomes [16]. Intervention was defined as “a treatment, whether for preventative or therapeutic reasons, an assessment or diagnostic tool or some other type of service or condition to which a patient might be exposed” [21]. Data from the included studies was coded by two authors (AM and RR; AM and CMS, respectively) using the three research questions.

Regarding the first research question; data was extracted according to the use of the ICF framework as described in the individual papers. Four different ways of operationalization of the ICF were the most typical descriptions used in a subset of the papers included: (a) structuring, (b) linking, (c) analysing, or (d) developing instruments or models, respectively. All the included papers´ description were categorised in these. *Structuring* was considered present, when data or outcomes were categorized, or themes or information from interviews was coded according to the ICF framework. However, in case structuring was followed by other use, data was extracted according to the latter. *Linking* was considered present, when health information (e.g. from questionnaires or interviews) was coded to specific ICF categories, based on linking rules, e.g. linking items in a questionnaire to categories in a core set [9, 22]. *Analysing* was considered present if the paper explicitly described that data were analysed, most commonly after data or information had been structured following the ICF framework. *Developing instruments or models* based on the ICF framework was the last reported usage.

Regarding the second research question the description of VR professionals (e.g. health professionals) involved were extracted, and a descriptive summary of their use of the ICF in order to inform the assessment of functioning was presented. Regarding the third question; the use of the ICF components (body function, body structures, activity and participation, environmental factors) and the ICF core set(s) was extracted based on the information provided in individual papers.

### Collating, Summarizing, and Reporting Results

A descriptive summary of the charted data was done independently by two authors on all the included papers. The coded data relevant to inform the three review questions were charted from each paper included and categorized according to content analysis [23–25]. Both deductive and inductive analyses were used, as the results were based on the description in the papers, e.g., of the pre-defined ICF components and core sets. The descriptive summary of the main results is presented in tables.

## Results

**Fig. 2:**
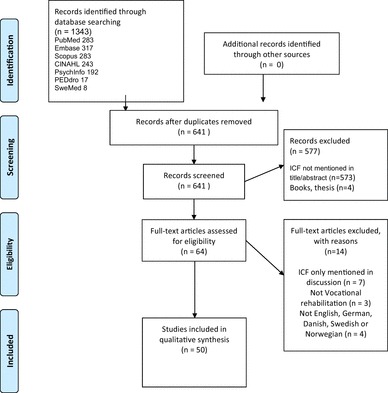
Flow diagram

In total 1343 papers were retrieved from seven databases, of which 702 duplicates were removed; thus, 641 papers were assessed for eligibility (Fig. 2). Sixty-four papers from these were read in full text of which 14 papers were excluded, mainly because the ICF was only mentioned in the introduction or discussion and lack of information on VR. Thus, 50 papers (25 qualitative and 25 quantitative) were included. No additional papers were included.

A descriptive summary of the included study characteristics is shown in Table 1. The ICF referral in papers within VR was found among Western countries, except one paper from Taiwan. Thus, nine papers were from Switzerland [10, 26–33] four were from the USA [34–37], three from Italy [38–40], two from Germany [41, 42], one from Canada [43], UK [44], Portugal [45], Turkey [46], Slovenia [47], Spain [48], Israel [49], and Taiwan [50]. Five papers were authored by an international [38, 51–54], three were from settings in Sweden [55–57], and three from Norway [58–60].

**Table 1:** Summary of basic characteristics of the included papers, aims, methods, and outcomes

Author	Year	Country	Setting	Intervention (yes/no) Study type	Population	Aims	Methods	Outcomes (primary/secondary)
Qualitative papers								
Abbott [55]	2011	Sweden	Orthopaedic Clinic, Karolinska University Hospital	Yes. Qualitative interview and self report scales	Lumbar fusion patients, N = 20	To describe within the context of ICF, patients’ experiences of post-lumbar fusion regarding low back problems, recovery and expectations of rehabilitation. To contrast with the content of outcome measures and the ICF low back pain core sets	Mixed method: Qualitative content analysis of semi-structured interviews 3–6 months after surgery and comparing ICF with questionnaires. ICF linking rules were used to code meaning units	Experiences with rehabilitation post-lumbar fusion. Expectation with outcome of rehabilitation ICF categories of all components
Aiachini [38]	2015	Team (Italy, USA, Switzerland)	Spinal unit at Rehabilitation hospital, Pavia	Yes. Focus group interviews	Patients with spinal cord injury (SCI), N = 24	To validate the comprehensive ICF Core Set for VR from the perspective of SCI patients To explore the aspects of functioning and health important to patients with SCI regarding RTW, and to examine to what extent these aspects are represented by the current version of VR core set	Focus group interviews 7 focus groups were digitally recorded and transcribed verbatim. The meaning condensation procedure was used for the data analysis Linking rules were used to code meaning units Adding the specific ICF Core Set for SCI in long-term context for not covered concepts	Concepts identified in the focus groups and their linking to ICF comprehensive core set for VR or not
Anner [26]	2012	Switzerland	Academy of Swiss Insurance Medicine, University Hospital Basel	No. Qualitative literature study	Sick-listed and persons unable to work (disability evaluation in medical reports)	To discuss potential benefits of the ICF to structure and phrase disability evaluation in the field of social insurance To describe core features of disability evaluation of the ICF across countries, and to address how and to what extent the ICF may be applied in disability evaluation	Qualitative method: a European comparison. Discussion of ICF (in 4 studies and in general) Reporting about work disability in social insurance	Core features for assessing work disability for medical experts
Bakker [65]	2006	Netherlands	Disability insurance, Centre of Healthcare Research, University Medical Centre Groningen	No. Literature study, consultations amongst experts	Disabled self-employed persons Four experts	To trace risk factors for disability amongst the self-employed To contribute to more evidence-based underwriting criteria for disability insurance	Literature study and consultation amongst four experts/researchers	Risk factors and medical characteristics in long-term disability in the employed and self-employed populations
Culler [34]	2011	USA	Rehabilitation Institute of Chicago	Yes. Qualitative interviews	Stroke survivors, employers, vocational specialists N = 10, 7, 21	To identify factors that facilitate or act as a barrier to RTW for stroke survivors	Qualitative methods: Interviews with stroke survivors about their RTW experience post stroke Survey with vocational specialists about barriers and facilitators of RTW based on their clinical practice. Interviews with employers experienced in interviewing	Barriers and facilitators to RTW for stroke survivors from three perspectives were illustrated Identified components were mapped based on the ICF
Dalemans [66]	2008	Netherlands	Health care, Zuyd University, Heerlen	No. Systematic review	Stroke survivors	To describe what is known in the literature about participation in working-age persons with aphasia after stroke	Systematic literature searching for the period 1960–2005 on participation: the performance of people in actual activities in social life domains through interaction with others in the context in which they live	Four social life domains (1) domestic life (2) interpersonal life (3) education and employment (4) community, civic, and social life, including religion, politics, recreation, and leisure
Daniel [44]	2009	UK	Stroke rehabilitation; Division of Health and Social Care Research	No. Systematic review of quantitative and qualitative studies	Stroke survivors	To identify the social consequences of stroke in working-aged adults, which might imply social needs to be addressed by health and social care services. Inform the development and evaluation of services for this group	Review of quantitative and qualitative studies identifying social consequences for working-aged adults with stroke using multiple search strategies	Prevalence of work after stroke Social consequences of stroke for working-aged adults
De Boer [61]	2009	Netherlands	Social insurance, Dutch Association of Insurance Medicine	Yes. Descriptive Survey and a questionnaire	Social Insurance Physicians (SIPs), N = 98	To investigate to what extent SIPs are familiar with the protocols, and to what extent they adhere to the principles of the expert- and practice-based protocols developed to conduct interviews with claimants for long-term incapacity for work	Mixed methods: Survey among experienced SIPs Qualitative study: comparison of the three protocols with each other and with ICF topics. Development of a questionnaire to elicit the adherence SIPs have to the protocols, their underlying principles and topics	Application of protocol(s) Training in and actual use. Construction of own protocol. Answers to questions noted in %, in total and per protocol
Desiron [67]	2013	Belgium	Department of Occupational, Environmental and Insurance Medicine, Leuven	No. Qualitative literature study	Persons with breast cancer	To identify a theoretical framework for occupational therapist (OT) intervention by questioning how OT models can be used in OT interventions in RTW of breast cancer patients	Literature searching: Research specific criteria derived from OT literature conceptual OT-model, multidisciplinary, referring to the ICF. Content analysis. Checking for breast cancer specific issues	OT models to facilitate RTW in breast cancer, matches between literature and care-models regarding RTW in breast cancer
Escorpizo [75]	2013	Team (USA, Germany, Switzerland)	Department of Physical Therapy, Louisiana State Uni-versity Health Sciences Center	No. Presentation paper	Aimed at persons to evaluate disability	To present the ICF as a standard in disability evaluation and to discuss the usefulness and challenges of the ICF when applied in disability evaluation including the ICF core set for VR.	Illustration of operationalizing the ICF in a hypothetical case of a construction worker who has chronic low back pain. Assessment of sample ICF categories and their integration in developing goals and planning intervention	Sample of ICF categories
Escorpizo [52]	2009	Team (Switzerland, Germany, Canada, Netherlands)	ICF Research Branch of the WHO Collaborating Center	No. Literature review	Aimed at researchers to select an appropriate questionnaire for a specific study question	To describe the content of self-report questionnaires that assess worker productivity and that are being used or could potentially be used in arthritis and other musculoskeletal conditions using the ICF as reference	Literature search, content examination and use of ICF categories as a reference for comparison of questionnaires	Meaningful concepts were identified and linked to the corresponding ICF category
Finger [29]	2014	Switzerland	Swiss Paraplegic Research, Nottwil	No. Case study (teaching case)	42-year-old teacher, who was on sick leave for 10 weeks due to non-specific low back pain N = 1	To illustrate an application of ICF-based tools in a multidisciplinary RTW program for patients with non-specific low back pain from the perspective of the physiotherapist To guide the rehabilitation process and facilitate team-based and physiotherapist goal setting and documentation	Assessment of employed discipline-specific clinical tests and measures taking into account the assigned ICF categories from the checklist. The team allocated the ICF categories included in the Rehabilitation Management-Sheet to the most appropriate long-term or short-term goals The team and patient agreed on the interventions that would target the specific goals and responsibilities	Categories included in the ICF-based tool (Rehabilitation Management-Sheet) and clinical tests and measures
Glassel [31]	2012	Switzerland	Swiss Paraplegic Research Nottwil	No. Case study	Patients with spinal cord injury. Aimed at VR professionals	To illustrate the systematic application of ICF-based documentation tools by using ICF Brief Core Sets in VR shown with a case example of a client with traumatic spinal cord injury (SCI)	Development of ICF-based documentation tools taking into account the ICF SCI Core Sets to facilitate the documentation and planning of rehabilitation services The tools include the ICF Assessment Sheet, ICF Categorical Profile, ICF Intervention Table and the ICF Evaluation Display	Presentation of ICF-based documentation tools: ICF Intervention Table and the ICF Evaluation Display of a client with SCI in a VR program
Glassel [30]	2011	Switzerland	Swiss Paraplegic Research Nottwil	No. Mixed-methods multicenter study, focus group design	Professionals in VR, N = 26	To explore the lived experiences of persons in VR with regard to functioning and contextual factors	Focus group interviews 7 focus groups yielding relevant concepts by 6 open-ended questions Linking to the ICF categories based on established linking rules: Transcription—Concept—ICF category—Qualitative analysis—Linking	Identified concepts related to the ICF components Classification of concepts with ICF as a reference
Hoefsmit [68]	2014	Netherlands	Department of Social Medicine, Maastricht University	No. Qualitative study	Employees, employers, occupational physicians, N = 14, 15, 4	To identify which and how environmental and personal factors support early RTW, and examine whether the ICF can be used to describe these factors	Interviews with employees, employers and OPs from multiple organisations with varying organisational sizes and types of industry such as healthcare and education. Qualitative data analysis partially based on the Qualitative Analysis Guide of Leuven	Factors that support employees’ early RTW and factors that can or cannot be described and classified using ICF coding
Homa [35]	2007	USA	Department of Rehabilitation and Counseling, University of Wisconsin-Stout	No. Overview, descriptive	Aimed at professionals in VR and researchers	To provide an overview of the ICF, highlight its applicability in job placement, and describe future possibilities for research and outcome measurement in VR	Use of the ICF framework in job placement as a template to organize client information, highlight strengths and limitations, and provide guidance for interventions in the placement process	Description of ICF used in job placement
Koolhaas [62]	2013	Netherlands	Department of Health Sciences, Community and Occupational Medicine, University Medical Center Groningen	Yes. Survey of perspectives	Workers, > 45 years, N = 3008	To understand the number and type of experienced ageing problems and obstacles to perform work tasks, retention factors to maintain work and support needs to continue working life in the next years among workers with and without a chronic health condition	Survey among workers in 9 companies. Classification using ICF Occupation was divided into four groups: executive, secretarial, policy and management Chronic health condition was defined as the subjective experience of long-term irreversible disease > 3 months	Problems and obstacles regarding work; age, gender, education, occupation, sector and whether the worker experienced a chronic health condition
Minis [69]	2009	Netherlands	Department of Occupation and Health, prevention and reintegration HAN University of Applied Sciences	No. Systematic review	Patients with neuromuscular diseases (NMD)	To identify health and contextual factors associated with employment in patients with NMD and to perform a best evidence synthesis	Literature search, extraction of factors related to employment status Results of the factor extraction related to employment were included in the scheme of Heerkens´ extended ICF model	Disease related factors, functions (physical, muscle power), personal factors (age, gender and education), work related personal factors (type of occupation, expressed interest in employment)
Sevilla [48]	2013	Spain	Electrical and Electronic Engineering Department, Universidad Publica de Navarra	No. Literature review	Persons with disabilities and intended users (employees, employers, or VR staff)	To propose a hierarchical model of accommodation assessment based on level of specificity of job activity	Literature review: Approach to the hierarchical model was tested against several case study scenarios to check its feasibility and completeness	Applications of the model to each of the cases´ core activities of occupations, such as: cook, office assistant, gardener, sewing machine operator, or real estate broker
Stergiou-Kita [43]	2013	Canada	Toronto Rehabilitation Institute, University Health Network	No. Systematic review to outline guidelines	Individuals with burn injuries (BI)	To gather evidence to develop a guideline for vocational evaluation following burn injuries (BI) To identify the key processes evaluators should follow and the key factors they should consider when completing such evaluations	Literature review; Searching in databases and websites Quality assessment: Using the ICF model and VR core sets and directed content analysis, key processes and factors were analysed and synthesized	Key factors and processes relevant to a vocational evaluation in relation to individual’s body functions, activity limitations and participation restrictions and personal and environmental supports to successful RTW
Trenaman [73]	2015	Canada/Switzerland	Department of Occupational Science and Occupational Therapy, University of British Columbia	No. Systematic review	Individuals with spinal cord injury	To review factors that are consistently and independently associated with employment outcomes in individuals with spinal cord injury To understand the magnitude of their influence	Literature search identified studies published 1952–2014. Data were categorized based on the ICF with each domain sub-categorized by modifiability	Modifiable and non-modifiable factors in the context of employment following SCI
van Velzen [63]	2011	Netherlands	Academic Medical Center, University of Amsterdam	Yes. Semi-structured interviews	Persons with acquired brain injury, N = 12	To describe the factors experienced by adults with moderate-to-severe acquired brain injury as either limiting or facilitating during the RTW process in order to give an advice about the VR process	Semi-structured interviews with 12 adults who were working before acquiring traumatic or non-traumatic brain injury (2–3 years earlier)	Aspects that were experienced as being important during the process of RTW after ABI
Vooijs [70]	2015	Netherlands	Amsterdam	No. Systematic review	People of working age with a chronic disease	To search systematically for disease-generic factors associated with either work retention or RTW in people of working age with a chronic disease	Literature search in databases, on synonyms of the terms: chronic disease, work retention and RTW	Factors associated with work participation for participants with a chronic disease (15–67 years)
Wasiak [54]	2007	USA, NZ, Netherlands	Liberty Mutual Research Institute for Safety, Center for Disability Research, Hopkinton, MA	No. Development study	Workers	To operationalize the conceptualization of RTW, which argues for an expanded awareness that encompasses 4 phases: off work, work reintegration, work maintenance and advancement	Review of existing instruments for their use as measures of RTW Where gaps in instrumentation were found, a wider search was done for instruments that could be adapted for use in RTW research	Use of measurement tools that do not capture a complete picture of workers’ RTW experiences
Young [36]	2010	USA	Liberty Mutual Research Institute for Safety, Center for Disability Research, Hopkinton, MA, USA	Yes. Quantitative and qualitative components	Occupationally injured workers after VR, N = 150	To determine post-RTW disability and functioning amongst occupationally injured workers To test the extent to which demographic and other variables relate to employment maintenance, and to document what workers believe determined their work continuation	Semi-structured in-depth interviews about participants´ post-VR RTW experiences regarding important factors determining their continuation of work	Factors experienced regarding RTW. Functional restrictions, activity-based-, or contextual- Factors differentiating those employed from those not
Quantitative papers								
Andelic [58]	2012	Norway	Hospital outpatient clinic, Oslo	No. Cross-sectional study	Patients with neck pain referred to the neck and back N = 249	To describe commonly reported self-determined functional problems in patients with neck pain E.g. problems with work participation To evaluate their fit to the components of the ICF	Self-reported functional problems on the Patient-Specific Functional Scale. The ICF was used as a tool for analysis. Meaningful concepts within the functional problems were identified, coded, and linked to second-level categories within the components of body functions, activities and participation. The ICF categories were presented by percentage of the total number of functional problems linked to the ICF	Functional problems fit with the ICF model; 13 meaningful ICF domains were identified: 4 domains in body function (= 12 underlying categories). 13 domains in activity and participation (= 31 underlying categories)
Chow [37]	2014	USA	Eight states	Yes. Longitudinal, 4 year 8-state multisite demonstration project, quasi-experimental design	Psychiatric disability out-patients: Severe and persistent mental illness, N = 1654 Referred by provider, self, family, word of mouth, newspaper ads	To evaluate the impact of an evidence-based approach to delivering employment services to individuals with psychiatric disabilities between 1996 and 2000 To compare those with/without reported work accommodations	Interview protocols, structured assessments, weekly recording, and detailed description of accommodations-summarized Effects assessed with models informed by ICF and other. Generalized linear model (number of hours of overtime work after job accommodation) and survival analysis (time until job shift/accommodation)	How job accommodations that are moderated by clinical and contextual factors are related to (1) average-monthly hours worked in competitive employment across multiple spells of employment? (2) the duration of job tenure across multiple spells of employment
Conclave [39]	2009	Italy	Nationwide, ordered by Italian Ministry of Labour and Social Policies	No. Experimental application of the ICF based method and development	Aimed for evaluation of Persons with disability (PwD)	To develop a nationwide ICF-based worker checklist To present the process and the results of ICF and Labour Policies Project with a special focus on the development of the checklist	Development of the dedicated ICF-based worker checklist on the basis of the ministerial schedule for the evaluation of PwD and the WHO’s ICF checklist, (a list of 128 ICF categories employed during ICF’s field trial) Standardised linking rules were followed to identify concepts contained in the ministerial schedule	Tools Two main tools have been produced by the ICF and labour policies project: the worker checklist and the protocol
de Beer [71]	2014	Netherlands	Department Occupation & Health, HAN University of Applied Sciences Nijmegen, The Netherlands	No. Systematic review	Persons with dyslexia or (specific) learning/reading disorder	To determine hindering and facilitating factors associated with participation in work of individuals with developmental dyslexia (DD), classified according to the dimensions of the ICF To explore and fully understand factors associated with work participation of adults with DD	Systematic literature search of quantitative or qualitative methodology, published after 1995. ICF-expanded with two subdivisions: one that made the environmental factors more work-related and one of personal factors. For data extraction: qualitative meta summary was used and the manifest frequency effect size (MFES) for each category	Effect size of factors between dyslexia or learning/reading disorder/disability and work The manifest frequency effect size is presented: calculated by dividing the number of all studies (that met the quality criterion) and in which a factor was found by the total number of studies
Escorpizo [75]	2011	Switzerland	Swiss Paraplegic Research, Notwill	No. Development study	Persons with spinal cord injury (SCI)	To develop a set of ICF-based SCI Participation and SCI Environment Domain Set and measurement instruments that intend to measure those domains, based on the ICF Core Sets for SCI	Merging of the ICF Core Set for SCI and categories from the ICF Core Set for VR	ICF categories based on the existing ICF core sets for SCI and VR
Escorpizo [33]	2011	Switzerland	Department of Health Sciences and Health Policy, Notwill	No. Development studies, international consensus conference	VR professionals and researchers	Presentation of five articles in an effort to advance our understanding and measurement of VR and RTW process	Different perspectives on ICF/VR	Distribution of ICF categories across ICF components and across studies. 3 core sets (2 SCI + 1 VR) and 6 instruments that measure environment and participation
Escorpizo [32]	2011	Switzerland	International survey	No. Internet-based survey with expert participants from six WHO Regions	VR professionals, (experts from 6 WHO Regions), N = 151	Survey the experts in the VR field with regard to what factors are considered important to patients participating in VR using the ICF as the language to summarize the results	Survey with VR experts. Question was related to a component of the ICF, responses linked to ICF	List of ICF categories that were considered to be important in the VR process
Ferrario [40]	2014	Italy	Occupational Medicine Department, Turin University	Yes. Cross- sectional study	Outpatients undergone heart transplantation, liver -, and kidney- and survived at least 12 months, N = 150	To provide evaluation of possible RTW and of fitness for specific and adequate tasks of surviving transplant recipients and to compare the results with the assessment of their actual employment status	ICF questionnaire; 10 questions were further applied to those who were employed at the time of the study. Questions regarding the following: time to RTW after surgery, jobs performed after RTW, part-time or fixed-shift job assignation, difficulties in performing the previous or the new job, possible periods of unemployment, satisfaction with the job gained after transplantation, the relations with the employer and the occupational physician, the support received	Comparison of working ability evaluation and employment status. Internal comparison among different organ recipients 61% of patients were in paid employment, 4% of students and housewives. 24% unemployed related or not to health conditions, 11% were retired
Finger [28]	2011	Switzerland	VR centres; 4 in Switzerland 1 in Germany	No. Cross-sectional multicenter study	Persons with various health problems > 18 years N = 152	To describe persons undergoing VR To identify the most common problems around work and in VR using the ICF	Case Record Form based on an extended version of the ICF Checklist containing 292 and SES	Categories from all four ICF components
Finger [27]	2014	Switzerland	VR centres	No. Development and validation study	Psychologist. Test-sample of patients 18–65 years, participating in VR N = 74	To develop an interviewer administered ICF-based questionnaire (WORQ) to assess functioning in VR To report preliminary psychometric evidence	Mixed methods including sophisticated statistical approach and qualitative content assessment. cat. from ICF VR-Core Sets, explorative Rasch-analysis and VR literature review. Questions were worded to assess identified ICF categories. WORQ was translated from English to German. Examination of psychometrics for the German version of WORQ	Items of WORQ, the ICF category measured
Kuijer [64]	2006	Netherlands	Centre for Rehabilitation, University Medical Centre Groningen	Yes. Cross. sectional study	Patients with chronic low back pain referred for multidisciplinary treatment N = 92	To explore which variables are related to work status according to ICF	Questionnaires (health, limitation), test of physical performance Logistic regression analysis was performed to explain work status (outcome) from the included variables of functioning	Work status, variables of functioning
Leyshon [76]	2008	Canada	University of Western Ontario	No. Literature review	Injured workers (musculoskeletal disorders most common)	To introduce an ICF-based new practice model of occupational rehabilitation ergonomics	Traditional model: Micro /macro-ergonomics have been defined as “the study and process of designing and/or modifying tools, materials, equipment, work spaces, tasks, jobs, products, systems, and environments to match the abilities, limitations, and social needs of human beings in the workplace”	Model, in order to better describe interventions, as interventions carried out in the workplace appear to be “very heterogeneous and ill-defined”
Linden [41]	2010	Germany	Inpatients, department of behavioural and psychosomatic medicine, Teltov	Yes. Cross-sectional, examination and interview	Patients admitted to the Department of Behavioral Medicine, N = 213	To examine the relationship between measures of capacity*, motivation and performance *inability to perform activities (i.e. dysfunctions)	Special clinical interview and questionnaires observer rating for Mental Disorders (Mini-ICF-APP), work performance Endicott Work Productivity Scale (EWPS), and volitional and motivational problems	Assessment of capacities (work-related attitudes, volition and motivation)
Martins [45]	2015	Portugal	Coimbra Health School, Physiotherapy Department	No. Explorative, cross-sectional study	Working-age persons with disabilities living in community dwelling settings. Severe limitations in mobility due to a chronic disease or injury, using a wheelchair for > 1 year, N = 149	To explore key indicators of social participation (life habits) of persons with disabilities, particularly related to work	Questionnaires: Attitudes Towards Disabled Persons Questionnaire, self-efficacy and the LIFE-H (77 items across 12 primary domains, including nutrition, fitness, personal care, communication, housing, mobility, responsibility, interpersonal relations, community, education, employment, and recreation)	Determinants for social participation, employment (self-efficacy, QoL)
Nilsing [56]	2012	Sweden	Hospital physicians and GPs, Ostergotland County	No. Comparative study	All new sick leave certificates during 2-week period in 2007 and 4-week period in 2009, N = 475501	To compare quality of sickness certificates between 2007 and 2009. (Differences between ICF-codes in 2007 and 2009)	Pearson’s chi2 and t-test was performed to test differences between variables	Quality in sickness certificates, description of functioning and prescriptions of early rehabilitation
Ptyushkin [47]	2011	Slovenia	Organisations granted to assess persons with disabilities and to operate their VR	No. Review, survey	Psychologists, social workers and occupational therapists	To review use of the ICF in VR and disability assessment	Review of the Slovenian law, survey, group and individual interviews. Nine of 13 organisations were surveyed totally or partially	Main advantages/dis-advantages and qualities/deficiencies; whether the ICF helps to establish a common language
Reichel [42]	2010	Germany	Inpatient rehabilitation centre, Bad Brückenau	Yes. Chart review	Patients with gastrointestinal diseases, N = 355	To link ICF to other specific instruments and compare with other predictors of rehabilitation outcomes	Screening files; Crohn’s Disease Activity Index variables were linked with ICF categories using linking rules	Variables linked with clinical improvement (decrease in Harvey-Bradshaw Index of ≥ 2 U) and VR success
Saltychev [46]	2013	Turkey	Turkish University Hospital	Yes. Retrospective cohort study	Patients with chronic musculoskeletal disorders, undergoing VR-evaluation, N = 32	To identify the most frequent functional limitations according to ICF	Each phrase from the patients’ electronic record that could potentially be interpreted as an ICF code was extracted	ICF codes categorized 141 different were identified with a preciseness of three or more digits
Sturesson [57]	2015	Sweden	Swedish Social Insurance Agency 146 different GPs, at 29 centres	Yes. Quality assessment, based on an intervention	Patients at primary health care centres, sickness certificates, N = 323	To evaluate the quality of sickness certificates issued in primary health care and examine if the patients’ or physicians’ gender influences	Evaluation was performed in accordance with the same criteria as in the national ‘Sick Leave Billion’	Sufficient information concerning the diagnosis, level of sick leave and time period for the sick leave
Varekamp [53]	2013	Netherlands/Germany	Current Health in Germany	No. Descriptive study, registers	Population 18–65 year, N = 35,574	To explore problems or solutions for workers with a chronic disease; from quantitative and qualitative research	Telephone surveys conducted from July 2008 to July 2010 among adults	Chronic disease, participation (work disability)
Wang [50]	2013	Taiwan	University College of Social Science	No. Survey, secondary analyses	Labour force with disability living at home, N = 2,909	To explore ICF factors association with employment in disabled	Survey: Life situation for disabled; secondary data analysis, regression model	Employment, type of disability and ICF category
Zeilig [49]	2012	Israel	Post-polio outpatient clinic. Tel-Hashomer	No. Data extraction from records	Patients with longstanding poliomyelitis (LSP), N = 123	To determine the effects of a number of social and functional variables as barriers or facilitators to work participation in persons with LSP	Review of the medical records. Employment defined as > = 20 h of regular remunerative activity	Employment; assistant devices for mobility, dependent for basic ADL associated with lower employment. Driving positive associations
Østerås [60]	2007	Norway	Ullensaker municipality	Yes. Survey	Seven birth cohorts, N = 1620	To provide measurement of population functional levels, assessment of reliability of a Norwegian scale based on ICF	Postal questionnaire in 2004	Instrument based on ICF-functional ability; derived from the activities/participation component
Aas [59]	2007	Norway	Community-based OT services	No. Cross-sectional postal survey	Clients in community health care, N = 168	To describe socio-demographic factors and the occurrence of diseases and disabilities among a representative sample of clients who were using community OT services	Community occupational therapy	Coded diagnoses -according to the International Classification of Primary Care (ICPC-2)
Aas [74]	2011	Norway	Cochrane Back Group	No. Systematic review	Adult workers with neck pain	To determine the effectiveness of workplace interventions compared to no treatment, usual care or other workplace interventions for adult workers with neck pain	Literature search, workers at work or absent from work. Workers with acute, sub-acute or chronic neck pain	Two main outcomes Pain relief and reduced sickness absence/RTW. Pain severity or pain prevalence

Although 32 of the papers were reviews, primarily from research settings (e.g. rehabilitation social medicine or physiotherapy departments) in Switzerland and the Netherlands; other VR settings in which the ICF was used were widespread, i.e. hospitals, rehabilitation centres, primary health care centres, and sickness certificate registration offices.

A minority of papers reported interventions within VR; only seven of the qualitative papers [34, 36, 38, 55, 61–63], and eight among the quantitative papers [37, 40–42, 46, 57, 60, 64]. Eleven papers were from health care and research settings in the Netherlands [61–71].

### How is the ICF Operationalized in Empirical Papers within VR?

In total 18 papers (36%) used the ICF as a framework for structuring of information: twelve of the qualitative papers [26, 27, 30, 44, 61, 62, 66, 68–70, 72–74], and six of the quantitative papers [29, 37, 42, 57, 64, 74]; e.g. relating information in sickness certificates to the ICF framework [57] (Table 2). As an example, one paper reported that the ICF was used for verifying data on claimants´ disabilities by comparing the information provided by the ICF and the bio-psycho model to see the extent of match [61].

**Table 2:** Summary of the included papers´ operationalization of ICF, persons involved in VR, and ICF components used

Author	Operationalization of ICF (i.e. as a framework for: structuring, linking, analysis or development)	Who are involved (stakeholders, patients)	ICF components used
Qualitative structuring			
Anner [26]	Framework to structure and phrase disability evaluation by use of ICF. Medical Evaluation of work disability. The ICF framework distinguishes the domains and their interaction but does not foresee a restricted causal relation	Researchers, medical evaluators of work disability	All components except personal factors
Dalemans [66]	Framework for categorizing. Search terms were derived from ICF. Aspects of domestic life, interpersonal interactions and relationships, education and employment, and community, civic, and social life were included	Researchers	Participation only In domestic life, inter-personal life, education, community, civic, and social life
Daniel [44]	Framework for categorizing. Defining social consequences according as those pertaining to the ICF domain of “participation”. Social consequences grouped into 5 domains reflecting the topics investigated: RTW, family relationships, sexual, finances, and social activities. Developing a standardized instrument, which takes into account specific needs of working-aged people. This scale should be in line with the ICF	Researchers	Participation only In work only
De Boer [61]	Framework and verification according to ICF categories. The topics that address a claimant’s disability were compared to ICF and a bio-psycho-social approach to see the extent of match	Researcher and social insurance physicians	All components
Escorpizo [75]	Framework. Description and use of ICF categories. Use of ICF as a language of disability, a common reference framework to provide disability criteria in determining functional and work capacity, and to help facilitate a common ground of understanding	Researchers	All components ICF generic set, core sets for VR and Disability Evaluation in Social Security
Finger [29]	Framework of structuring. Application and comparison of ICF-based tools the Rehabilitation Management Sheet, the Work Rehabilitation Questionnaire (WORQ, the generic and brief core set of low back pain). ICF structures used to facilitate communication between stakeholders, to help structure rehabilitation plans and for setting goals, and clarifying team roles	Researcher and stakeholders: rehabilitation physician, a physiotherapist, a psychologist and a vocational counsellor	All components Except personal factors Core set of low back pain (LBP)
Glassel [31]	Framework for a systematic application of ICF-based documentation tools by using ICF Core Sets in VR. Use of the ICF Core Sets in VR allows a comprehensive assessment	Researcher and VR team OT, physical therapist, nurses, vocational Counsellor, social worker, physician, and psychologist	All components Core set for VR
Hoefsmit [68]	Framework for description of environmental and personal factors regarding support of employees´ RTW. Professionals´ use of the ICF	Researcher and persons interviewed: 14 employees, 15 employers and 4 OPs from multiple organisations (healthcare and education)	All components. Except personal factors
Koolhaas [62]	Framework for categorization. ICF used for classification and comparing the workers’ perspectives	Researcher	All components
Minis [69]	Framework, ICF used as a structure for factor extraction indicative for association with employment status from studies. Factors related to health state, work and other environmental and personal factors is needed to improve care and services by allied health professionals and organisations involved in the (re-) integration process	Researcher	All components
Trenaman [73]	Framework for categorization. Factors categorized based on the ICF with each domain sub-categorized by modifiability	Researchers	All components
Vooijs [70]	Framework for categorization. Factors associated with work participation were categorized according to ICF. Various disease-generic factors are associated with work participation, of which most of the reported factors are independent of diagnosis	Researchers	All components
Qualitative linking			
Aiachini [38]	Framework for linking. Validation of core set for VR. Concepts were linked to ICF categories according to established linking rules. 70% of 90 categories in VR core set were found	Spinal cord injury patients, two health professionals linked the concepts to ICF	All components. Comprehensive core set for VR
Escorpizo [52]	Framework for linking, ICF used as a reference to describe and compare the contents of these questionnaires: Health and Work Q., Work Role Functioning Q. Rheumatoid Arthritis-Work Instability Scale, Health and Labour Q	Researchers	All components Health and Work Q. the only including environmental and personal factors
Glassel [30]	Framework for linking. Reference to ICF categories according to established linking rules	Researcher	All components
Qualitative analysing			
Abbott [55]	Framework for analysing interviews. Based on ICF a qualitative content analysis of semi-structured interviews post-surgery was performed. ICF was applied to identify and code meaningful units, which were compared with the ICF related content of the Oswestry Disability Index, SF-36, EQ5D and the ICF LBP core sets	Patients, researchers	All components Core set for low back pain
Culler [34]	Framework for analysing interviews. Components identified in 3 perspectives (patients, vocational experts and employers) were illustrated and mapped onto the ICF coding	10 stroke survivors, 21 vocational specialists, 7 employers (experienced in interviewing persons with disabilities and with authority to make hiring decisions)	All components. Impairments of body, activity limitations to participation. Restrictions by environmental and personal factors
Stergiou-Kita [43]	Framework for data analysis. Utilized as guiding frameworks during data analysis. ICF focused more specifically on identifying key domains or factors and failed to capture the processes relevant to a rigorous evaluation	Researcher	All components
Van Velzen [63]	Framework for the interview and the analysis	Researchers	All components
Young [36]	Framework for analysing interviews. Results were interpreted using the health and health-related domains from the ICF. Interviews were conducted to inquire about participant’s post-VR RTW experiences. Coding of the qualitative data and analysis was conducted in tandem	Researchers and post VR participants	All components
Qualitative development			
Bakker [65]	Framework for developing a risk assessment model, with a strong focus on personal and environmental factors, as it will affect claim behaviour. The model will bring the current medical model at the underwriting stage more in line with the social model at claim stage	Researchers	All components Focus on environmental and personal factors in addition to medical data
Desiron [67]	Framework used to identify elements in OT models. Research specific criteria derived from OT literature (conceptual OT multidisciplinary model referring to the ICF)	Researcher	All components. Identified elements: functional, medical, RTW
Homa [35]	Framework for development of interview format informed by the ICF structure. Used in job placement as a template to organize client information, highlight strengths and limitations, and provide guidance for interventions	Researcher	All components Except personal factors
Sevilla [48]	Framework for development of a model of which the levels of activity can be cross-walked to the ICF	Researcher	All components The new model include more than ICF
Wasiak [54]	Framework for developmental conceptualization of RTW. Using the ICF to inform our thinking and coding structure, conceptualizing phase-based RTW outcomes and categorization in ‘tasks and actions’, ‘contextual’ or ‘process driven’. Awareness of RTW encompassing four phases: off work, work reintegration, work maintenance and advancement	Researchers	All components
Quantitative structuring			
Chow [37]	Framework for categorization and description on how limitations in functioning and the environment are related to employment outcomes	Research team; trained interviewers; project staff members	All components Personal characteristics
Finger [27]	Framework for structuring. ICF core set basis for developing an instrument. Participants commented on the usability	Professionals, 25 patients, vocational counsellors, and a work reintegration specialist	All components Except personal factors Core set
Kuijer [64]	Framework for classification. Variables classified according to the ICF	Research assistant; 2 physiotherapists (PT); PT/movement scientist, trained, certified and experienced	All components Part 1, functioning and disability Part 2, contextual factors
Reichel [42]	Framework for categorization. Linking each meaningful concept and objective with the most precise ICF category	Physicians specialized in gastroenterological rehabilitation	Body functions/body structures only
Sturesson [57]	Framework for categorization and verifying the information of sickness certificates. The assessment of Swedish Social Insurance Agency (SSIA) has to verify that the information clarifies a logical link between diagnoses, impairment of body function and activity limitation (the ‘DFA chain’). The vocabulary and definitions in the DFA chain are in accordance with the ICF	Independent insurance specialist from the SSIA, educated and trained to assess the quality	Impairment of body function, limitation of activity only
Aas [74]	Framework for categorization. ICF terminology was used to classify the intervention components. ICF could have contributed to a conceptual frame of reference based on common terminology	Researchers	All components
Quantitative linking			
Conclave [39]	Framework for structuring followed by linking Italian legislative procedures to the ICF domains and categories, and adding standard ICF checklist categories. The ICF-based worker checklist is composed of 183 ICF categories	Professionals in job placement of persons with disabilities. Participants: 895 in Basic ICF training, 552 in Advanced	All components
de Beer [71]	Framework for linking, coding. The factors from all studies coded on the two-level classifications of ICF. Frequency and consistency in hindering or facilitating made visible by use of ICF categories	Research team	All components Work-related activities, participation, environmental and personal factors
Escorpizo [75]	Framework for structuring followed by linking. Items of measurement instruments were linked to the ICF core sets, applying the linking rules	Researcher, two coders	All components except body factors Activities, participation and environment components. Comprehensive ICF Core Set for SCI, VR
Escorpizo [33]	Framework for structuring followed by linking. Linking between 3 core sets of 6 questionnaires assessing environment and participation by two independent researchers. Merging ICF categories	International team of researchers	All components Except personal factors
Escorpizo [32]	Framework for linking. ICF applying published linking rules; responses were listed and frequency analysis was performed	Responders (151), experts from 47 countries, random sample of professions, WHO regions, countries	All components
Quantitative analysing			
Andelic [58]	Framework, tool for analysis. Linking of self-reported problems related to neck pain to domains of the ICF	Research team	All components except environmental factors. Domains loading on the activities and participation
Nilsing [56]	Framework for analysis. Free text on functioning was analysed deductively using the ICF framework and placed into categories	Researchers and an adjudicator. Consensus meeting between the researchers and adjudicator	Body and activity only (Sensations of pain or emotional functions. Walking or handling stress)
Wang [50]	Framework for analysis. Dependent and independent variables based on ICF, and their operational definition were used for coding; e.g. 0 = not employed/no, 1 = employed/yes	Researcher	All components
Ferrario [40]	Framework for analysis. ICF questionnaire; used the ICF to evaluate working ability of transplant recipients to provide the occupational physicians a standardized procedure to suggest the best possibility of re-employment in close co-operation with the patient	Occupational physician of the Occupational Medicine Department, researcher	All components
Finger [28]	Framework for analysis. Identification of the most common problems around work and in VR. Examine the frequency and rate problems based on the extended ICF checklist (the ICF Checklist version 2.1a)	Health professionals	All components Except personal factors
Saltychev [46]	Framework for coding followed by analysis of comparison. Descriptions of functional limitations were converted to ICF codes, and the most frequent were compared with the ICF Checklist and VR core sets	Multi-professional team. (Specialist in physical and rehab. medicine, rehab. planner, psychologist and the patients)	All components Except personal factors Core set for VR
Zeilig [49]	Framework for analysis. Barriers and facilitators of working participation defined according to the ICF categories. Levels of function were then analysed for correlation to the vocational status	Researcher	Body functions and activities only. Focus on mobility in regard to employment status
Quantitative development			
Leyshon [76]	Framework, basis for a new model. Discussion of opportunities to use this model in researching outcomes of ergonomic interventions. Illustrate how the ICF framework could be applied to a worker with a low back disorder	Researcher	All components
Linden [41]	Framework for evaluation. Use of mini ICF to assess its clinical relevance. Correlations made with other instruments. Functions, capacities and participation are not linear but interactive, as known from occupational psychology	Researcher	All components
Martins [45]	Framework for evaluation. To explore correlations between social participation, employment and personal factors such as self-efficacy and attitudes towards disability	Researcher	All components except body functions and activity
Ptyushkin [47]	Framework for development of questionnaire. Subject for questionnaire: VR professionals´ opinions about ICF. (How would you define the ICF? What is the ICF for you? and ‘In your opinion, what is the purpose of the ICF’) Integration of the ICF into the Slovenian VR and Employment of Persons with Disabilities Act made the use of ICF obligatory	45 professionals involved in VR (Psychologists, social workers, technologists, OTs, physicians, education counsellor, rehabilitation counsellor)	Body functions component only
Varekamp [53]	Framework for evaluation. Understanding and considering health-related problems at work and finding solutions; ICF used as a model to explain work disability. Prevalence of chronic medical conditions (non-communicable diseases) is strongly related to age	Researcher	All components Focus on environmental and organisational factors
Østerås [60]	Framework for development. ICF used as basis for development of national questionnaire (Norwegian Function Assessment Scale)	Researcher	Activities/participation components
Aas [59]	Framework for development. ICF used as basis for questionnaire in survey of impairment, activity limitations, and participation restrictions (e.g. participation in ordinary working life) 9 OTs from 4 municipal areas tested the questionnaire on 18 clients	Occupational therapists (OT) and clients	Body, activity and participation components

In total eight papers (15%) used the ICF as a framework for linking between ICF categories and e.g. items in questionnaires: three qualitative papers [31, 38, 52], and five quantitative papers [32, 33, 39, 71, 75]; e.g. of Italian legislative procedures to the ICF [39], of factors coded on the second- level ICF classifications [71], of items to the core set and following the linking rules [32, 33, 75]. As an example, one paper aimed at merging an ICF core set for a specific health-related condition (spinal cord injury) to the categories of the VR core set [75]. Another paper identified the concepts within the functional problems which were coded, and linked to ICF categories, or to the categories of the VR core set [38].

The analysis was performed according to the ICF framework in 12 papers (24%): five qualitative papers [34, 36, 43, 55, 63], e.g. listing of the respondents´ answers followed by frequency analysis according to the relevant ICF domains [55] and seven quantitative papers [28, 40, 46, 49, 50, 56, 58]; e.g. rating and analysing problems regarding work [28], and extraction of phrases from a patients´ electronic record that could potentially be interpreted as an ICF category [46]. One of the quantitative papers analysed levels of function and how it correlated with vocational status [49].

The ICF was used as a framework for the development of an instrument or a new model for various aspects within VR in 12 papers (24%) : five qualitative papers [35, 48, 54, 65, 67]; e.g. a model relating the levels of activity to the ICF [48], and a model explaining work disability by health-related problems at work [53]. A paper concluded that the ICF may contribute by informing our thinking of RTW and work maintenance by conceptualizing phase-based RTW outcomes [54]. Seven quantitative papers reported use of the ICF for development [41, 45, 47, 53, 59, 60, 76]; e.g. relating with other questionnaires used in VR [41], and use of the ICF core sets for developing a questionnaire for description of workplace accommodation [60].

### Who are Involved and How Does the ICF Inform the Professionals´ Assessment of Functioning in VR?

Four papers described involvement of patients and researchers [34, 55] or patients and health professionals [38, 61]. Two papers described involvement of professionals, employers, and employees as informants [68], medical professionals as evaluators of work disability and researchers [26], respectively. A majority (32) of papers were reviews involving solely the authors (researchers): twenty of the qualitative papers, and eleven of the quantitative papers, respectively. Two papers involved a research team, interviewers and project staff [37], researchers and an adjudicator [56], respectively. Seven papers involved researcher and numerous VR professionals [32, 39, 40, 42, 46, 47, 59], e.g. psychologists, social workers, technologists, occupational therapists, occupational physicians, education counsellor, rehabilitation counsellor. Two papers involved health professionals and patients [28], and solely health professionals [27], respectively. A paper described all professionals involved in rehabilitation research (experienced physiotherapists, certified physiotherapist/movement scientist, research assistant) [64], another paper involved independent insurance specialists, who were trained to assess the quality of information in sickness certificates [57].

### How the ICF Inform Assessment of Functioning

Regarding to what extent the ICF informed professionals´ assessment of functioning; several papers reported discussions on the ICF´s applicability for VR, service delivery, and RTW support. As examples were papers reporting on potential benefits of the ICF: to structure and phrase disability evaluation in the field of social insurance [26], on tracking risk factors for disability amongst the self-employed [65], highlight its applicability in job placement [35], and to identify the most common problems around work and in VR.

One paper concluded that a questionnaire based on the ICF proved to be a “useful framework that can be used for research but also by occupational physicians in their usual practice after specific training” [29]. A paper reported on an expert survey on use of the ICF as the language to summarize the results in VR [32]. Another paper concluded, that although the procedure using the ICF was “complex, time-consuming, and requires specific training of the staff involved in its use”; the occupational physicians were provided with a standardized procedure to evaluate working ability and suggest re-employment for transplant recipients [40].

A paper described how VR professionals used the ICF to guide assessment in the job placement process and used the appropriate ICF domains and categories as a template to determine what specific information needed to be obtained, and how to organize it in a systematic way. Thus, an interview format informed by the ICF structure enabled the professionals to highlight the needs for assessment information [35].

### Criticism of the ICF

One paper involving several health and non-health professionals concluded, that disadvantages of the ICF are the “complicated terminology, perceived subjectivity of the assessor in coding” and that ‘it is too bulky’ [47]. Another paper described factors that support employees’ early RTW and reported that some factors cannot be described and classified using the existing coding system of the ICF [68].

### Which of the ICF Components and Core Sets are Considered When Functioning is Evaluated in VR?

Except in 10 papers all the ICF components were described. Two papers commented on personal factors, despite the fact they are part of the ICF there are no categorizations [29, 35]. Only two papers described the component participation [44, 66]. One paper described all components but environmental factors [58]. Six papers evaluated the body functions component only [42, 47, 49, 56, 57, 59], and three papers reported on all components except body functions or participation components [45, 60, 75].

Seven papers used the ICF core set [27, 29, 30, 38, 46, 51, 55], among which four studies reported on the core set for VR [30, 38, 46, 72]. The VR core set was used for validation of another ICF core set [38], development of ICF-based documentation tools [30], comparison of the most frequent ICF coding of functional limitations with the ICF Checklist and VR core sets [46].

## Discussion

The ICF was primarily used in Western VR contexts. The ICF used as a framework was the most prevalent operationalizing of ICF (18 papers), whereas linking, analysing and developing appeared in 8, 12 and 12 papers respectively. As 32 of the 50 included papers were reviews the predominant profession involved in ICF were researchers. Among the original papers no single profession stood out as particularly ICF users. In general the ICF enabled the various professions involved in VR in a structured way to obtain relevant need assessments and communicate this across professions. The majority (40) of papers described all factors, which support the bio-psycho-social approach. However, it was not clear if the ICF was suitable as an instrument for goal setting and evaluation as merely single papers mentioned these properties. Moreover, the ICF was criticised for being time consuming. Unexpectedly four papers described the ICF components body and acidity only, despite participation and environmental factors seem inseparable from VR. The VR core set was not the primary tool when functioning was evaluated within VR.

Compared to the findings in another review where qualitative papers only constituted a tenth [10], and despite some papers with a mixed study designs were defined as qualitative in this review, the number of qualitative and quantitative papers was more balanced in this review.

The ICF defines functioning as the interaction between an individual and that individual’s environmental and personal factors; accordingly a paper illustrated the problems of functioning in a person with low back pain by use of the framework [75].

The ICF is seen as a useful tool for describing, comparing and contrasting information from outcome measures and clinical patient reports across diagnoses, settings, languages and countries [77]. A review showed that linking health and health-related information to the ICF is a useful way to apply the ICF in research [77].

Evaluation of functioning is relevant early in VR [8], and this review found several presentations of the applicability for VR and use of the ICF to examine and measure VR processes and outcomes. The findings show that the ICF was useful in providing a clear description of the consequences of diseases, and of the factors that can be described using the ICF coding, which may potentially support the VR professionals, e.g. factors that support employees’ early RTW [68]. The ICF can help VR professionals gain a more precise understanding of the impact of disability on individuals’ ability to perform life tasks or activities. Thus, the ICF might contribute to a more informative description in multi-professional assessments, because healthcare professionals have different perspectives on the health-care process [57]. However, a paper concluded that in primary care there seem to be a lack of knowledge about the ICF, and that increased cooperation between GPs and other health-care professions may require learning as well as a change of attitudes [57].

Furthermore, an ICF-based questionnaire regarding time to RTW, work difficulties, job satisfaction, and work relations was reportedly useful for occupational physicians assessing patients after transplant procedures [40]. Thus, the ICF framework provided an effective evaluation of possible RTW and capabilities of these patients, who had undergone transplants and survived at least 12 months. However, the procedure of for assessment of self-reported work ability was reported as complex and required specific training of the staff involved [40].

The present review illustrates how the ICF may support development of questionnaires [59, 60], like e.g. the Work Rehabilitation Questionnaire (WORQ). The WORQ has proven to be a valuable instrument within VR [27], e.g. as to support the physiotherapist´s role within the rehabilitation team by enhancing transparency in goal setting and intervention planning across disciplines [29].

Although the ICF is a reasonable starting point in efforts to harmonize terminologies [33], the framework is also criticised for limitations. This scoping review reported on the ICF components only. However, each of the components (except for personal factors) is further divided into domains and underlying categories providing more detail of a component. The ICF coding system is intended to describe a person’s functioning at a specific time, in that person’s normal circumstances and environment. Qualifiers are built into the coding system to indicate the magnitude of the impairment, limitation or restriction for each category.

A review on the use of ICF in outcome measures used within VR identified that a third of categories were related to body functions [10]. This review also identified some papers solely reporting on body functions, which is not representing a bio-psycho-social approach. It should be recognized that the ICF is limited with respect to comprehensive descriptions of work disability, e.g. the cause why a person is not able to work is an important part of disability evaluation. The ICF however, cannot describe causal relationship [26], and a solution may be to use the ICF combined with other instruments, which can reveal causal relations. It was pointed out that although the framework includes personal factors, they cannot be classified in the ICF [68]. This is a limitation of the framework, as e.g. motivation is important to consider when making prognosis of work ability and RTW. Personal factors also include an individual’s lifestyle, habits, social background, education, life events, race/ethnicity, sexual orientation, and coping mechanisms [43]. Work participation relies on both personal and environmental factors, which in addition to the medical data, affect functioning and participation, e.g. a paper concluded these factors affect claim behaviour [65]. Factors that are likely to be emphasized in a VR setting are within the components: activities, participation and environmental factors [35]. A Cochrane review found a lack of interventions targeting the ICF-domains: attitudinal and social environment [74]. However, this review found only a few papers lacking the environmental factors.

Unexpectedly, the review revealed a limited use of the ICF VR core sets. These include environmental factors that may prove to be useful when disability evaluation and work capacity is being assessed. However, a validation study of the comprehensive VR core set concluded, that it was insufficient from a sole physiotherapist perspective, there was a need for additional ICF categories. Although the VR core set was considered useful to clarify responsibilities and for communication in a multidisciplinary setting, it was too comprehensive for mono-disciplinary use of physiotherapist [78]. The core sets in general were not recognized to provide an exhaustive list but rather the minimum number of categories to be assessed [8]. Therefore practice may supply the VR core set with other instruments in order to fully assess functioning.

## Strengths and Limitations

One strength was the inclusion of both qualitative and quantitative papers from multiple settings and countries. Furthermore, the scoping review format offers an overview of study findings in a field where the knowledge is still limited.

The reviewers experienced difficulties in study selection, despite the method by two reviewers and how to ensure eligibility criteria is a limitation. In a scoping review the extracted data is based on information provided in individual papers without critical assessment, which is a limitation despite no scope of synthesizing evidence. Furthermore, the categories of operationalization may not be mutual exclusive.

The sixth and optional stage of involving relevant stakeholders was not included but may have contributed with other VR professionals´ views [15].

### Implications for Practice within VR

This review confirm challenges with the use of the ICF: e.g. it cannot infer causality in disability [8] and not categorize personal factors. The content of VR varies widely among countries because of differing insurance policies and disability attitudes; e.g. in Slovenia the ICF for work assessment was made obligatory but the lack of interface between the ICF and policies on VR was a challenge [47]. The ICF may be used to ensure comprehensiveness of evaluation in study populations with chronic diseases [46]. Furthermore, the framework may cover all relevant aspects of disability and may encourage the VR professionals to draw a holistic picture [26]. The ICF “corresponds closely to this ecological systems approach and could help rehabilitation practitioners more specifically and precisely identify those subsystems or environmental factors that have an impact on successful job placement” [35]. The ICF may be combined with existing measures and incorporated in daily practice [31].

### Implications for Future Research in Work Disability and VR

Our findings revealed that the ICF has been applied in different settings and for different purposes, which has important implications for future research. In order to ensure comparability across studies and robust testing of hypotheses the use of the ICF needs to be clarified. Furthermore, how data are collected, assessed and classified based is lacking in the field of VR. Hence, research on the practical utility of the ICF across different assessment instruments is crucially needed to inform a feasible framework development in VR.

Although the ICF provides a framework to evaluate contextual factors, this review finds there is a gap between the knowledge of the impact of personal factors and actual assessment within VR and more research is needed.

## Conclusions

The scoping review revealed use of the ICF within the field VR in 50 papers, and in various settings; e.g. hospitals, rehabilitation centres, primary health care centres, sickness certificate registration offices, and research departments. The operationalization of the ICF was described in four ways: for structuring information, linking of categories or content, analysis according to the ICF framework, or development of instruments or models based on the ICF.

A majority of papers were reviews and involved researchers only, whereas different stakeholders and VR professionals were involved in the interventions. The components of the ICF that depict functioning and disability were largely incorporated in the VR research. This observation points to the benefit of using a common set of ICF components to inform the selection of set of measurement instruments. Such a process would lead to a single set of standardized measures looking at similar outcomes and make comparability across studies possible However, more research is needed to develop and validate instruments measuring relevant domains including personal factors and to standardize and ease the VR professionals´ use of the ICF.

## Electronic supplementary material

Below is the link to the electronic supplementary material.

Supplementary material 1 (DOCX 50 KB)
