# Supplementary material for: International Classification of Functioning, Disability and Health in Vocational Rehabilitation: A Scoping Review of the State of the Field
Source: J Occup Rehabil. 2018 Jun 5;29(2):241–73. doi: 10.1007/s10926-018-9788-4 (PMC6531389; doi:10.1007/s10926-018-9788-4)
Supplement: Supplementary file 1 — Supplementary material 1 (DOCX 50 KB) [file 10926_2018_9788_MOESM1_ESM.docx]

# Appendix A. Search history

**Results**

**PubMed 28-09-2015 283 references**

((((((((((((("Rehabilitation, Vocational"[Mesh]) OR Vocational Rehabilitation*) OR occupational rehab*) OR work rehab*) OR job rehab*) OR "Sick Leave"[Mesh]) OR ("Sick Leave" OR "Disability Leave" OR "sickness absence" OR sicklist*)) OR "Return to Work"[Mesh]) OR ("Return to Work" OR "Back to Work")) OR ("work ability" OR "work disability" OR "work re-integration" OR "re-entry")) OR "Employment"[Mesh]) OR Employ*)) AND (("International Classification of Functioning, Disability and Health"[Mesh]) OR international classification of functioning disability and health OR ("International Classification of Functioning Disability and Health") OR ("icf"[Title/Abstract] OR "icidh"[Title/Abstract] OR "icidh2"[Title/Abstract] OR "icide-2"[Title/Abstract]) OR ("ICF classification"[Title/Abstract] OR "ICF code"[Title/Abstract] OR "ICF Core Set"[Title/Abstract] OR "ICF Core Sets"[Title/Abstract] OR "ICIDH"[Title/Abstract] OR "International Classification of Impairments Disabilities AND Handicaps"[Title/Abstract]) OR "international classification impairment*" OR "international classification impairment* disabilit*" OR "international classification impairment* disabilit* handicap*") Filters: Publication date from 2000/01/01; English; Danish; Norwegian; Swedish; German

**Results**

**Embase 28-09-15 317 references**

**#44** AND (**'article'**/it OR **'article in press'**/it OR **'review'**/it)

**426**

**#44**

**#26** AND **#42** AND ([danish]/lim OR [english]/lim OR [german]/lim OR [norwegian]/lim OR [swedish]/lim) AND [2000-2015]/py

**469**

**#43**

**#26** AND **#42**

**555,998**

**#42**

**#27** OR **#28** OR **#29** OR **#30** OR **#31** OR **#32** OR **#33** OR **#34** OR **#35** OR **#37** OR **#38** OR **#41**

**9,371**

**#41**

**'work ability'** OR **'work re-integration'** OR **'re-entry'** OR **'work disability'**

**531,548**

**#38**

**employ***

**55,345**

**#37**

**'employment'**/exp

**9,149**

**#35**

**'return to work'** OR **'back to work'**

**1,857**

**#34**

**'return to work'**/exp

**7,860**

**#33**

**'medical leave'** OR **'sick leave'** OR **'disability leave'** OR **'sickness absence'** OR **sicklist***

**4,093**

**#32**

**'medical leave'**/exp

**19**

**#31**

**job** NEXT/1 **rehab***

**346**

**#30**

**work** NEXT/1 **rehab***

**1,497**

**#29**

**occupational** NEXT/1 **rehab***

**9,921**

**#28**

**vocational** NEXT/1 **rehabilitation***

**8,762**

**#27**

**'vocational rehabilitation'**/exp

**6,146**

**#26**

**#21** OR **#22** OR **#23** OR **#24** OR **#25**

**260**

**#25**

**international** NEXT/2 **classification** NEXT/2 **impairment***

**3,051**

**#24**

**'icf classification'**/exp OR **'icf classification'** OR **'icf code'**/exp OR **'icf code'** OR **'icf core set'**/exp OR **'icf core set'** OR **'icf core sets'**/exp OR **'icf core sets'** OR **'icidh'**/exp OR **'icidh'** OR **'international classification of functioning disability and health'**/exp OR **'international classification of functioning disability and health'** OR **'international classification of impairments, disabilities, and handicaps'**/exp OR **'international classification of impairments, disabilities, and handicaps'**:ab,ti

**5,049**

**#23**

**'icf'** OR **'icich'** OR **'icidh2'**:ab,ti

**3,052**

**#22**

**'icf classification'**/exp OR **'icf classification'** OR **'icf code'**/exp OR **'icf code'** OR **'icf core set'**/exp OR **'icf core set'** OR **'icf core sets'**/exp OR **'icf core sets'** OR **'icidh'**/exp OR **'icidh'** OR **'international classification of functioning disability and health'**/exp OR **'international classification of functioning disability and health'** OR **'international classification of impairments, disabilities, and handicaps'**/exp OR **'international classification of impairments, disabilities, and handicaps'**

**1,646**

**#21**

**'international classification of functioning, disability and health'**/exp

**413**

**#20**

**#6** AND **#19** AND ([danish]/lim OR [english]/lim OR [german]/lim OR [norwegian]/lim OR [swedish]/lim) AND [2000-2015]/py

**553,274**

**#19**

**#7** OR **#8** OR **#9** OR **#10** OR **#11** OR **#12** OR **#13** OR **#14** OR **#15** OR **#16** OR **#17** OR **#18**

**531,548**

**#18**

**employ***

**55,345**

**#17**

**'employment'**/exp

**9,371**

**#16**

**'work ability'** OR **'work re-integration'** OR **'re-entry'** OR **'work disability'**

**9,149**

**#15**

**'return to work'** OR **'back to work'**

**1,857**

**#14**

**'return to work'**/exp

**7,860**

**#13**

**'medical leave'** OR **'sick leave'** OR **'disability leave'** OR **'sickness absence'** OR **sicklist***

**4,093**

**#12**

**'medical leave'**/exp

**19**

**#11**

**job** NEXT/1 **rehab***

**346**

**#10**

**work** NEXT/1 **rehab***

**1,497**

**#9**

**occupational** NEXT/1 **rehab***

**9,921**

**#8**

**vocational** NEXT/1 **rehabilitation***

**8,762**

**#7**

**'vocational rehabilitation'**/exp

**6,146**

**#6**

**#1** OR **#2** OR **#3** OR **#4** OR **#5**

**260**

**#5**

**international** NEXT/2 **classification** NEXT/2 **impairment***

**3,051**

**#4**

**'icf classification'**/exp OR **'icf classification'** OR **'icf code'**/exp OR **'icf code'** OR **'icf core set'**/exp OR **'icf core set'** OR **'icf core sets'**/exp OR **'icf core sets'** OR **'icidh'**/exp OR **'icidh'** OR **'international classification of functioning disability and health'**/exp OR **'international classification of functioning disability and health'** OR **'international classification of impairments, disabilities, and handicaps'**/exp OR **'international classification of impairments, disabilities, and handicaps'**:ab,ti

**5,049**

**#3**

**'icf'** OR **'icich'** OR **'icidh2'**:ab,ti

**3,052**

**#2**

**'icf classification'**/exp OR **'icf classification'** OR **'icf code'**/exp OR **'icf code'** OR **'icf core set'**/exp OR **'icf core set'** OR **'icf core sets'**/exp OR **'icf core sets'** OR **'icidh'**/exp OR **'icidh'** OR **'international classification of functioning disability and health'**/exp OR **'international classification of functioning disability and health'** OR **'international classification of impairments, disabilities, and handicaps'**/exp OR **'international classification of impairments, disabilities, and handicaps'**

**1,646**

**#1**

**'international classification of functioning, disability and health'**/exp

| **Results**  **Scopus 29-09-15 283 references**  ( ( TITLE-ABS-KEY ( **"international classification of functioning disability and health"**  OR  **"icf"**  OR  **"icidh"**  OR  **"icidh2"**  OR  **"icidh-2"** ) )  OR  ( TITLE-ABS-KEY ( **"icf classification"**  OR  **"icf code"**  OR  **"icf core set"**  OR  **"icf core sets"** ) )  OR  ( TITLE-ABS-KEY ( **"international classification of impairments disabilities and handicaps"** ) ) )  AND  ( ( TITLE-ABS-KEY ( **"Vocational Rehabilitation*"**  OR  **"occupational rehab*"**  OR  **"work rehab*"** ) )  OR  ( TITLE-ABS-KEY ( **"job rehab*"**  OR  **"Sick Leave"**  OR  **"Disability Leave"**  OR  **"sickness absence"**  OR  **sicklist*** ) )  OR  ( TITLE-ABS-KEY ( **"Return to Work"**  OR  **"Back to Work"** ) )  OR  ( TITLE-ABS-KEY ( **"work ability"**  OR  **"work disability"**  OR  **"work re-integration"**  OR  **"re-entry"**  OR  **"Employment"** ) ) )  AND  ( LIMIT-TO ( LANGUAGE , **"English"** )  OR  LIMIT-TO ( LANGUAGE ,  **"German"** )  OR  LIMIT-TO ( LANGUAGE ,  **"Norwegian"** )  OR  LIMIT-TO ( LANGUAGE ,  **"Swedish"** ) )  AND  ( LIMIT-TO ( PUBYEAR ,  **2015** )  OR  LIMIT-TO ( PUBYEAR ,  **2014** )  OR  LIMIT-TO ( PUBYEAR ,  **2013** )  OR  LIMIT-TO ( PUBYEAR ,  **2012** )  OR  LIMIT-TO ( PUBYEAR ,  **2011** )  OR  LIMIT-TO ( PUBYEAR ,  **2010** )  OR  LIMIT-TO ( PUBYEAR ,  **2009** )  OR  LIMIT-TO ( PUBYEAR ,  **2008** )  OR  LIMIT-TO ( PUBYEAR ,  **2007** )  OR  LIMIT-TO ( PUBYEAR ,  **2006** )  OR  LIMIT-TO ( PUBYEAR ,  **2005** )  OR  LIMIT-TO ( PUBYEAR ,  **2004** )  OR  LIMIT-TO ( PUBYEAR , **2003** )  OR  LIMIT-TO ( PUBYEAR ,  **2002** )  OR  LIMIT-TO ( PUBYEAR ,  **2001** )  **Results**  **CINAHL 28-09-2015 243 references**   \|  \| [Search ID#](javascript:__doPostBack('ctl00$ctl00$FindField$FindField$historyControl$ReorderHistoryLink','')) \| **Search Terms** \| **Search Options** \| **Actions** \| \| --- \| --- \| --- \| --- \| --- \| \|  \| S23 \| S5 AND S19 \| **Limiters** - Published Date: 20000101-20151231  **Narrow by Language:**- german  **Narrow by Language:**- english  **Search modes** - Boolean/Phrase \| [**View Results**](javascript:__doPostBack('ctl00$ctl00$FindField$FindField$historyControl$HistoryRepeater$ctl00$linkResults','')) (243)  [**View Details**](javascript:showShDetails(%22ctl00_ctl00_FindField_FindField_historyControl_ctrlPopup%22,%20%22S23%22);)  [**Edit**](http://web.a.ebscohost.com.ez.statsbiblioteket.dk:2048/Legacy/Views/UserControls/EHOST/) \| \|  \| S22 \| S5 AND S19 \| **Limiters** - Published Date: 20000101-20151231  **Narrow by Language:**- english  **Search modes** - Boolean/Phrase \| [**View Results**](javascript:__doPostBack('ctl00$ctl00$FindField$FindField$historyControl$HistoryRepeater$ctl01$linkResults','')) (230)  [**View Details**](javascript:showShDetails(%22ctl00_ctl00_FindField_FindField_historyControl_ctrlPopup%22,%20%22S22%22);)  [**Edit**](http://web.a.ebscohost.com.ez.statsbiblioteket.dk:2048/Legacy/Views/UserControls/EHOST/) \| \|  \| S21 \| S5 AND S19 \| **Limiters** - Published Date: 20000101-20151231  **Search modes** - Boolean/Phrase \| [**View Results**](javascript:__doPostBack('ctl00$ctl00$FindField$FindField$historyControl$HistoryRepeater$ctl02$linkResults','')) (244)  [**View Details**](javascript:showShDetails(%22ctl00_ctl00_FindField_FindField_historyControl_ctrlPopup%22,%20%22S21%22);)  [**Edit**](http://web.a.ebscohost.com.ez.statsbiblioteket.dk:2048/Legacy/Views/UserControls/EHOST/) \| \|  \| S20 \| S5 AND S19 \| **Search modes** - Boolean/Phrase \| [**View Results**](javascript:__doPostBack('ctl00$ctl00$FindField$FindField$historyControl$HistoryRepeater$ctl03$linkResults','')) (253)  [**View Details**](javascript:showShDetails(%22ctl00_ctl00_FindField_FindField_historyControl_ctrlPopup%22,%20%22S20%22);)  [**Edit**](http://web.a.ebscohost.com.ez.statsbiblioteket.dk:2048/Legacy/Views/UserControls/EHOST/) \| \|  \| S19 \| S6 OR S7 OR S8 OR S9 OR S10 OR S11 OR S12 OR S13 OR S14 OR S15 OR S16 OR S17 OR S18 \| **Search modes** - Boolean/Phrase \| [**View Results**](javascript:__doPostBack('ctl00$ctl00$FindField$FindField$historyControl$HistoryRepeater$ctl04$linkResults','')) (102,157)  [**View Details**](javascript:showShDetails(%22ctl00_ctl00_FindField_FindField_historyControl_ctrlPopup%22,%20%22S19%22);)  [**Edit**](http://web.a.ebscohost.com.ez.statsbiblioteket.dk:2048/Legacy/Views/UserControls/EHOST/) \| \|  \| S18 \| employ* \| **Search modes** - Boolean/Phrase \| [**View Results**](javascript:__doPostBack('ctl00$ctl00$FindField$FindField$historyControl$HistoryRepeater$ctl05$linkResults','')) (90,403)  [**View Details**](javascript:showShDetails(%22ctl00_ctl00_FindField_FindField_historyControl_ctrlPopup%22,%20%22S18%22);)  [**Edit**](http://web.a.ebscohost.com.ez.statsbiblioteket.dk:2048/Legacy/Views/UserControls/EHOST/) \| \|  \| S17 \| (MH "Employment+") \| **Search modes** - Boolean/Phrase \| [**View Results**](javascript:__doPostBack('ctl00$ctl00$FindField$FindField$historyControl$HistoryRepeater$ctl06$linkResults','')) (30,236)  [**View Details**](javascript:showShDetails(%22ctl00_ctl00_FindField_FindField_historyControl_ctrlPopup%22,%20%22S17%22);)  [**Edit**](http://web.a.ebscohost.com.ez.statsbiblioteket.dk:2048/Legacy/Views/UserControls/EHOST/) \| \|  \| S16 \| "work ability" OR "work re-integration" OR "work disability" \| **Search modes** - Boolean/Phrase \| [**View Results**](javascript:__doPostBack('ctl00$ctl00$FindField$FindField$historyControl$HistoryRepeater$ctl07$linkResults','')) (744)  [**View Details**](javascript:showShDetails(%22ctl00_ctl00_FindField_FindField_historyControl_ctrlPopup%22,%20%22S16%22);)  [**Edit**](http://web.a.ebscohost.com.ez.statsbiblioteket.dk:2048/Legacy/Views/UserControls/EHOST/) \| \|  \| S15 \| job re-entry OR ( "return to work" OR "back to work" ) \| **Search modes** - Boolean/Phrase \| [**View Results**](javascript:__doPostBack('ctl00$ctl00$FindField$FindField$historyControl$HistoryRepeater$ctl08$linkResults','')) (5,371)  [**View Details**](javascript:showShDetails(%22ctl00_ctl00_FindField_FindField_historyControl_ctrlPopup%22,%20%22S15%22);)  [**Edit**](http://web.a.ebscohost.com.ez.statsbiblioteket.dk:2048/Legacy/Views/UserControls/EHOST/) \| \|  \| S14 \| job re-entry \| **Search modes** - Boolean/Phrase \| [**View Results**](javascript:__doPostBack('ctl00$ctl00$FindField$FindField$historyControl$HistoryRepeater$ctl09$linkResults','')) (4,039)  [**View Details**](javascript:showShDetails(%22ctl00_ctl00_FindField_FindField_historyControl_ctrlPopup%22,%20%22S14%22);)  [**Edit**](http://web.a.ebscohost.com.ez.statsbiblioteket.dk:2048/Legacy/Views/UserControls/EHOST/) \| \|  \| S13 \| (MH "Job Re-Entry") \| **Search modes** - Boolean/Phrase \| [**View Results**](javascript:__doPostBack('ctl00$ctl00$FindField$FindField$historyControl$HistoryRepeater$ctl10$linkResults','')) (4,038)  [**View Details**](javascript:showShDetails(%22ctl00_ctl00_FindField_FindField_historyControl_ctrlPopup%22,%20%22S13%22);)  [**Edit**](http://web.a.ebscohost.com.ez.statsbiblioteket.dk:2048/Legacy/Views/UserControls/EHOST/) \| \|  \| S12 \| "Sick Leave" OR "disability leave" OR "sickness absence" OR sicklist* \| **Search modes** - Boolean/Phrase \| [**View Results**](javascript:__doPostBack('ctl00$ctl00$FindField$FindField$historyControl$HistoryRepeater$ctl11$linkResults','')) (3,590)  [**View Details**](javascript:showShDetails(%22ctl00_ctl00_FindField_FindField_historyControl_ctrlPopup%22,%20%22S12%22);)  [**Edit**](http://web.a.ebscohost.com.ez.statsbiblioteket.dk:2048/Legacy/Views/UserControls/EHOST/) \| \|  \| S11 \| (MH "Sick Leave") \| **Search modes** - Boolean/Phrase \| [**View Results**](javascript:__doPostBack('ctl00$ctl00$FindField$FindField$historyControl$HistoryRepeater$ctl12$linkResults','')) (2,786)  [**View Details**](javascript:showShDetails(%22ctl00_ctl00_FindField_FindField_historyControl_ctrlPopup%22,%20%22S11%22);)  [**Edit**](http://web.a.ebscohost.com.ez.statsbiblioteket.dk:2048/Legacy/Views/UserControls/EHOST/) \| \|  \| S10 \| job rehab* \| **Search modes** - Boolean/Phrase \| [**View Results**](javascript:__doPostBack('ctl00$ctl00$FindField$FindField$historyControl$HistoryRepeater$ctl13$linkResults','')) (142)  [**View Details**](javascript:showShDetails(%22ctl00_ctl00_FindField_FindField_historyControl_ctrlPopup%22,%20%22S10%22);)  [**Edit**](http://web.a.ebscohost.com.ez.statsbiblioteket.dk:2048/Legacy/Views/UserControls/EHOST/) \| \|  \| S9 \| work rehab* \| **Search modes** - Boolean/Phrase \| [**View Results**](javascript:__doPostBack('ctl00$ctl00$FindField$FindField$historyControl$HistoryRepeater$ctl14$linkResults','')) (1,093)  [**View Details**](javascript:showShDetails(%22ctl00_ctl00_FindField_FindField_historyControl_ctrlPopup%22,%20%22S9%22);)  [**Edit**](http://web.a.ebscohost.com.ez.statsbiblioteket.dk:2048/Legacy/Views/UserControls/EHOST/) \| \|  \| S8 \| occupational rehab* \| **Search modes** - Boolean/Phrase \| [**View Results**](javascript:__doPostBack('ctl00$ctl00$FindField$FindField$historyControl$HistoryRepeater$ctl15$linkResults','')) (1,276)  [**View Details**](javascript:showShDetails(%22ctl00_ctl00_FindField_FindField_historyControl_ctrlPopup%22,%20%22S8%22);)  [**Edit**](http://web.a.ebscohost.com.ez.statsbiblioteket.dk:2048/Legacy/Views/UserControls/EHOST/) \| \|  \| S7 \| vocational rehabilitation* \| **Search modes** - Boolean/Phrase \| [**View Results**](javascript:__doPostBack('ctl00$ctl00$FindField$FindField$historyControl$HistoryRepeater$ctl16$linkResults','')) (4,765)  [**View Details**](javascript:showShDetails(%22ctl00_ctl00_FindField_FindField_historyControl_ctrlPopup%22,%20%22S7%22);)  [**Edit**](http://web.a.ebscohost.com.ez.statsbiblioteket.dk:2048/Legacy/Views/UserControls/EHOST/) \| \|  \| S6 \| (MH "Rehabilitation, Vocational+") \| **Search modes** - Boolean/Phrase \| [**View Results**](javascript:__doPostBack('ctl00$ctl00$FindField$FindField$historyControl$HistoryRepeater$ctl17$linkResults','')) (4,825)  [**View Details**](javascript:showShDetails(%22ctl00_ctl00_FindField_FindField_historyControl_ctrlPopup%22,%20%22S6%22);)  [**Edit**](http://web.a.ebscohost.com.ez.statsbiblioteket.dk:2048/Legacy/Views/UserControls/EHOST/) \| \|  \| S5 \| S1 OR S2 OR S3 OR S4 \| **Search modes** - Boolean/Phrase \| [**View Results**](javascript:__doPostBack('ctl00$ctl00$FindField$FindField$historyControl$HistoryRepeater$ctl18$linkResults','')) (2,431)  [**View Details**](javascript:showShDetails(%22ctl00_ctl00_FindField_FindField_historyControl_ctrlPopup%22,%20%22S5%22);)  [**Edit**](http://web.a.ebscohost.com.ez.statsbiblioteket.dk:2048/Legacy/Views/UserControls/EHOST/) \| \|  \| S4 \| international classification impairment* \| **Search modes** - Boolean/Phrase \| [**View Results**](javascript:__doPostBack('ctl00$ctl00$FindField$FindField$historyControl$HistoryRepeater$ctl19$linkResults','')) (111)  [**View Details**](javascript:showShDetails(%22ctl00_ctl00_FindField_FindField_historyControl_ctrlPopup%22,%20%22S4%22);)  [**Edit**](http://web.a.ebscohost.com.ez.statsbiblioteket.dk:2048/Legacy/Views/UserControls/EHOST/) \| \|  \| S3 \| "ICF classification" OR "ICF code" OR "ICF Core Set" OR "ICF Core Sets" OR "International Classification of Impairments, Disabilities, and Handicaps" \| **Search modes** - Boolean/Phrase \| [**View Results**](javascript:__doPostBack('ctl00$ctl00$FindField$FindField$historyControl$HistoryRepeater$ctl20$linkResults','')) (283)  [**View Details**](javascript:showShDetails(%22ctl00_ctl00_FindField_FindField_historyControl_ctrlPopup%22,%20%22S3%22);)  [**Edit**](http://web.a.ebscohost.com.ez.statsbiblioteket.dk:2048/Legacy/Views/UserControls/EHOST/) \| \|  \| S2 \| ( "International Classification of Functioning, Disability, and Health" ) OR "icf" OR ( "icidh" OR "icidh2" ) \| **Search modes** - Boolean/Phrase \| [**View Results**](javascript:__doPostBack('ctl00$ctl00$FindField$FindField$historyControl$HistoryRepeater$ctl21$linkResults','')) (2,406)  [**View Details**](javascript:showShDetails(%22ctl00_ctl00_FindField_FindField_historyControl_ctrlPopup%22,%20%22S2%22);)  [**Edit**](http://web.a.ebscohost.com.ez.statsbiblioteket.dk:2048/Legacy/Views/UserControls/EHOST/) \| \|  \| S1 \| (MH "International Classification of Functioning, Disability, and Health") \| **Search modes** - Boolean/Phrase \| [**View Results**](javascript:__doPostBack('ctl00$ctl00$FindField$FindField$historyControl$HistoryRepeater$ctl22$linkResults','')) (1,705)  [**View Details**](javascript:showShDetails(%22ctl00_ctl00_FindField_FindField_historyControl_ctrlPopup%22,%20%22S1%22);)  [**Edit**](http://web.a.ebscohost.com.ez.statsbiblioteket.dk:2048/Legacy/Views/UserControls/EHOST/) \| |  |  |  |  |
| --- | --- | --- | --- | --- | --- | --- | --- | --- | --- | --- | --- | --- | --- | --- | --- | --- | --- | --- | --- | --- | --- | --- | --- | --- | --- | --- | --- | --- | --- | --- | --- | --- | --- | --- | --- | --- | --- | --- | --- | --- | --- | --- | --- | --- | --- | --- | --- | --- | --- | --- | --- | --- | --- | --- | --- | --- | --- | --- | --- | --- | --- | --- | --- | --- | --- | --- | --- | --- | --- | --- | --- | --- | --- | --- | --- | --- | --- | --- | --- | --- | --- | --- | --- | --- | --- | --- | --- | --- | --- | --- | --- | --- | --- | --- | --- | --- | --- | --- | --- | --- | --- | --- | --- | --- | --- | --- | --- | --- | --- | --- | --- | --- | --- | --- | --- | --- | --- | --- | --- | --- | --- | --- | --- | --- |
| **PsycInfo 29-09-15 Results**  **192 references**  Set#: S1  Searched for: international classification of functioning disability and health*  Databases: PsycINFO  Results: 1091°  Set#: S2  Searched for: ab("icf" OR "icidh" OR "icidh2" OR "icidh-2") OR ti("icf" OR "icidh" OR "icidh2" OR "icidh-2")  Databases: PsycINFO  Results: 1230°  Set#: S3  Searched for: ab("icf classification" OR "icf code" OR "icf core set" OR "icf core sets" OR "international classification of impairments disabilities and handicaps") OR ti("icf classification" OR "icf code" OR "icf core set" OR "icf core sets" OR "international classification of impairments disabilities and handicaps")  Databases: PsycINFO  Results: 178°  Set#: S4  Searched for: ab(international classification impairment*) OR ti(international classification impairment*)  Databases: PsycINFO  Results: 533°  Set#: S5  Searched for: (international classification of functioning disability and health*) OR (ab("icf" OR "icidh" OR "icidh2" OR "icidh-2") OR ti("icf" OR "icidh" OR "icidh2" OR "icidh-2")) OR (ab("icf classification" OR "icf code" OR "icf core set" OR "icf core sets" OR "international classification of impairments disabilities and handicaps") OR ti("icf classification" OR "icf code" OR "icf core set" OR "icf core sets" OR "international classification of impairments disabilities and handicaps")) OR (ab(international classification impairment*) OR ti(international classification impairment*))  Databases: PsycINFO  Results: 1836°  Set#: S6  Searched for: SU.EXACT.EXPLODE("Vocational Rehabilitation")  Databases: PsycINFO  Results: 6456*  Set#: S8  Searched for: "Vocational Rehabilitation*"  Databases: PsycINFO  Results: 10297*  Set#: S9  Searched for: "occupational Rehab*"  Databases: PsycINFO  Results: 821°  Set#: S10  Searched for: "work Rehab*"  Databases: PsycINFO  Results: 277°  Set#: S11  Searched for: "job Rehab*"  Databases: PsycINFO  Results: 9°  Set#: S12  Searched for: SU.EXACT("Employee Leave Benefits")  Databases: PsycINFO  Results: 806°  Set#: S13  Searched for: "sick leave" OR "disability leave" OR "sickness absence" OR "sicklist*"  Databases: PsycINFO  Results: 1697°  Set#: S14  Searched for: "return to work" OR "back to work" OR "work ability" OR "work disability" OR "work reintegration" OR "job re-entry" OR "re-entry"  Databases: PsycINFO  Results: 3761°  Set#: S15  Searched for: employ*  Databases: PsycINFO  Results: 215372*  Set#: S16  Searched for: SU.EXACT.EXPLODE("Vocational Rehabilitation") OR "Vocational Rehabilitation*" OR "occupational Rehab*" OR "work Rehab*" OR "job Rehab*" OR SU.EXACT("Employee Leave Benefits") OR ("sick leave" OR "disability leave" OR "sickness absence" OR "sicklist*") OR ("return to work" OR "back to work" OR "work ability" OR "work disability" OR "work reintegration" OR "job re-entry" OR "re-entry") OR employ*  Databases: PsycINFO  Results: 224167*  Set#: S17  Searched for: s5 AND s16  Databases: PsycINFO  Results: 201°  Set#: S18  Searched for: ((s5 AND s16) AND la.exact("English" OR "Swedish" OR "Norwegian" OR "German" OR "Danish")) AND yr(2000-2015)  Databases: PsycINFO  Results: 192°° |  |  |  |  |
|  |  |  |  |  |
| **SveMed . 29-09-15 Results**  **8 references**  Nr Söksträng Antal träffar    2 "icf" OR "icidh" OR "icidh2" OR "icidh-2" OR "icf classification" OR "icf code" OR "icf core set" OR "icf core sets" 91  5 international classification of functioning disability and health 55  8 exp:"international classification of functioning disability and health" Limits: mesh_en:"international classification of functioning disability and health" 17  10 international classification of impairments* 1  11 #2 OR #5 OR #8 OR #10 124  13 Vocational Rehabilitation OR occupational rehab* OR work rehab* OR job rehab* OR "Sick Leave" OR "Disability Leave" OR "sickness absence" OR sicklist* OR "Return to Work" OR "Back to Work" OR "work ability" OR "work disability" OR "work re-integration" OR "re-entry" OR Employ* 15  15 exp:"return to work" 33  16 exp:"vocational rehabilitation" 490  17 exp:"Employment" 1567  18 exp:"sick leave" 986  19 #13 OR #15 OR #16 OR #17 OR #18 2611  20 #11 AND #19 8 |  |  |  |  |
| **Pedro - Physiotherapy Evidence Database 02-10-15 Results**  **17 references**  ICF and rehab* =14  ICF and work = 1  ICF and employ* =1  ICIDH* =1 |  |  |  |  |
| \| **In total 1343 references** \|  \|  \|  \|  \| \| --- \| --- \| --- \| --- \| --- \| |  |  |  |  |
|  |  |  |  |  |
